# Pediatric Exposures to Neurotoxicants: A Review of Magnetic Resonance Imaging and Spectroscopy Findings

**DOI:** 10.3390/diagnostics12030641

**Published:** 2022-03-05

**Authors:** Kim M. Cecil

**Affiliations:** Departments of Radiology and Pediatrics, Cincinnati Children’s Hospital Medical Center, University of Cincinnati College of Medicine, Cincinnati, OH 45229, USA; kim.cecil@cchmc.org

**Keywords:** morphometry, magnetic resonance imaging, functional magnetic resonance imaging, diffusion imaging, magnetic resonance spectroscopy, environment, heavy metals, air pollution, lead exposure, flame retardants, environmental tobacco smoke, pesticides

## Abstract

Heavy metals, including lead and manganese, air pollution, pesticides, environmental tobacco smoke, and flame retardants are among the known and suspected environmental neurotoxicant exposures examined with magnetic resonance imaging (MRI)-based studies of pediatric populations. Many studies feature morphological changes associated with the exposures while others employ magnetic resonance spectroscopy, diffusion imaging, task-based, and resting state functional magnetic resonance imaging to reveal abnormal metabolic concentrations, white matter disorganization, and atypical patterns of activation. Some studies follow pregnant women and their offspring throughout the lifespan with collection of individual specimens as exposure biomarkers. Others innovatively make use of public databases to obtain relevant exposure biomarkers while taking advantage of these studies in their efforts to monitor developmental features in large, population-based, imaging cohorts. As exposures to neurotoxicants in the womb and throughout childhood have life-long impacts on health and well-being, the importance of these innovative neuroimaging investigations is ever increasing.

## 1. Introduction

Neuroimaging studies of pediatric populations exposed to environmental chemicals is an emerging area of research with the objective of uniquely informing the field of public health about brain structure, organization, and function while helping to unravel the contribution of environmental chemicals to the increased prevalence of neurodevelopmental disorders. Environmental epidemiologists traditionally employ cognitive and neurobehavioral assessments to assess the health effects environmental chemicals exert on pediatric brain development. Chemicals with adverse effects are classified as neurotoxicants as they are toxic substances introduced into the environment by human activity that harm the central nervous system. Established neurotoxicants include the heavy metals, air pollutants, some classes of pesticides, and tobacco smoke. As thousands of chemicals are introduced annually, there are limitations on exposure assessments and biomonitoring methods. Suspected neurotoxicants include flame retardants, perfluoroalkyl substances, phthalates, phenols, and polyphenols [1].

Pediatric populations are exposed to known and suspected toxicants from their mothers during gestation and with breast feeding, neonatally from products used for care in the medical setting, postnatally from the contaminated environment (air, soil, and water) that also taints the food ingested along with the products used in food storage and preparation (e.g., cans, pans, and other containers), and with transference from parental occupational exposures brought into the home. The ever-increasing use of consumer products in our homes, such as electronics, furniture, carpeting, cosmetics, etc., also expose children to neurotoxicants. 

This manuscript highlights only a few of the magnetic resonance imaging (MRI)-based studies of pediatric populations exploring associations with known or suspected neurotoxicant exposures. While some of these studies evaluate populations with high levels of a single neurotoxicant, for the majority, these exposures do not arise from an “accidental discharge” but instead occur from continual exposure to wide-spread, low-level mixtures found in ordinary living. Several studies employ typically developing, longitudinal pregnancy and birth cohorts with individually monitored low-level, ubiquitous exposures of environmental chemicals. Exposures may be present for a short-term at a key stage in pediatric development or persist as chronic exposures throughout childhood. Bioaccumulation may also contribute to the exposure effects. Traditional study designs quantitatively assess individual exposures employing biomarkers determined from whole blood, serum, urine, hair, and toenails to use in comparison models for statistical analyses. Moreover, analyses from large cohorts having neuroimaging originally designed for other purposes, such as the Adolescent Brain Cognitive Development Study (ABCD Study) are also included in this review. For these types of studies, individual participant biomarkers are often absent. However, using the individual participant home address, research teams can model estimates of lead exposure risk from community public records that detail housing age. Furthermore, spatial temporal models can take air quality monitoring data and home addresses to produce individual estimates of air pollutants for select periods of time. Exposure models have strengths and limitations, just as the neuroimaging acquisitions and analyses have their benefits and pitfalls. While the overarching objective of environmental epidemiology studies is to understand how environmental chemicals impact the brain by assessing cognition (e.g., full scale intelligence quotient (FSIQ)), reading skills, motor skills) and behaviors including mental health outcomes (attention disorders, anxiety, depression, etc.), those incorporating neuroimaging are also indirectly benefiting other research disciplines and clinical care. The variability found in clinical and research data collected within neuroimaging studies of diseases or in normal development may arise in part from the unrecognized adverse effects of maternal and childhood exposures to neurotoxicants. While a neurotoxicant exposure may alter structural volume by a relatively insignificant amount, such as 2% in an individual, when this is applied across a population, it can have a large impact [2,3].

## 2. Heavy Metals

Heavy metals are the most extensively studied neurotoxicants as they are naturally occurring elements with their incorporation into products used since ancient times. Lead was incorporated into commercial products beginning around 4000 B.C. with water pipes, pottery, coins, paint, and more recently with gasoline. Manganese (Mn) usage in pesticides and a gasoline additive may yield excess exposure and accumulation in humans. While Mn plays an essential role as a trace element in bone mineralization, cellular protection from damaging free radical species, protein and energy metabolism, excess in vivo concentrations have been shown to produce cognitive and neuromotor effects. Ingested Mn is under tight homeostatic control; however, inhaled Mn bypasses the biliary excretion mechanism and crosses the blood brain barrier via several pathways including facilitated diffusion and active transport. Mn enters the brain following nasal inhalation through axonal transport from the olfactory bulb to the cerebral cortex. Mn accumulates in iron-rich brain regions of the basal ganglia: caudate, putamen, globus pallidus, substantia nigra, and subthalamic nuclei of the brain. In contrast, there is no biological requirement for lead. Lead mimics calcium for selective passage across the blood brain barrier and long-term storage within the bone with reabsorption into the blood stream when calcium is needed, such as with bone growth and producing further deposition within the brain. This section features select MRI studies evaluating the effects of lead and Mn absorption during childhood. A summary table highlighting elements of the study design and findings for investigations associating imaging findings with lead assessments (Table 1) is provided.

### 2.1. Lead Exposure and Intelligence

Cognitive and behavioral assessments of individuals exposed to lead have demonstrated adverse changes in brain function. Several cohort studies from around the world individually demonstrated the adverse effect of environmental lead exposure on FSIQ, as it is sensitive for assessing cognitive function and can be reliably measured during childhood. When combining data into a pooled analysis from existing cohort studies for over 1300 children followed from birth until 10 years of age, Lanphear and co-authors found using a covariate-adjusted log-linear model, a 6.7 FSIQ point decrement associated with an increase in concurrent blood lead concentrations from 2.4 to 30 μg/dL, with the steepest slope for FSIQ loss found for the first 10 μg/dL [18]. Effects this large warranted changes in brain structure, organization, and function, however, lead itself cannot be directly or indirectly detected by MRI methods.

#### 2.1.1. Initial Magnetic Resonance Spectroscopy Evaluations of Children with Elevated Blood Lead Concentrations

Two small studies were among the first pediatric environmental epidemiology studies investigating the effects of childhood lead exposure. Trope et al. examined the frontal cortex of lead-exposed children using high-resolution magnetic resonance spectroscopy (MRS) for evidence of metabolic differences. The initial case-control report pointed to a reduction of N-acetyl-aspartate (NAA) to creatine (Cr) ratio within the middle frontal cortex of a 10-year-old boy with elevated blood lead concentrations (e.g., 51 μg/dL at 38 months) compared to his healthy 9-year-old cousin living in the same household [4]. A subsequent small cohort study with 16 children having at least one blood lead concentration exceeding 23 μg/dL before the age of 5 years replicated the case-control study finding [5].

#### 2.1.2. Multimodal Neuroimaging of the Cincinnati Lead Study

Building upon the Trope studies, researchers sought to replicate and expand the findings with a multimodal neuroimaging study of the Cincinnati Lead Study (CLS) cohort. The CLS enrolled pregnant women from 1979 to 1984 who lived in urban, inner-city neighborhoods with historically high rates of childhood lead poisoning to form a longitudinal pregnancy and birth cohort that was followed for several decades to evaluate the effects of childhood lead exposure on development. A reduction in FSIQ (mean cohort and standard deviation (SD) 87 (11.3)), poorer postural balance and motor outcomes in childhood [19], increased delinquency rates in adolescence [20], and higher criminal arrests in adulthood [21] (mean number of arrests 5.63 (SD 8.27) in adulthood through 2013, with 78% of the cohort having at least 1 arrest) were all associated with childhood blood lead concentrations and adjusted for confounding variables. The CLS enrolled 305 newborns who were followed-up for developmental and neurobehavioral assessments quarterly through age 5 years, and semi-annually from age 5 to age 6.5 years. Four subsequent visits were at 10 years of age, between the ages of 15–17 years, 19–24 years, and 27–33 years. Blood lead concentrations were obtained prenatally, at birth, every three months to 60 months of age, every 6 months from 60 to 78 months of age, during adolescence between 15 and 17 years of age, and during adulthood between 27 and 33 years of age. The mean childhood blood lead concentration for the cohort, 13 μg/dL (range 5–37 μg/dL), was often used for comparisons with outcomes and derived using twenty-three blood lead concentration assessments collected between 3 and 78 months of life. The peak lead exposure occurred between 2 and 3 years of age. During the first five years of life, at least one of the quarterly blood lead assessments exceeded 10 μg/dL for 99% of the cohort [7]. By adolescence, the mean blood lead concentrations for the cohort were 2.8 μg/dL (SD 1.3). By adulthood, 77% CLS participants had blood lead concentrations below the limit of detection with the remaining having a mean concentration of 1.1 μg/dL (SD 0.6). In 1991, the US Centers for Disease Control established a blood lead concentration of 10 μg/dL as the lowest level of concern. The value was eventually lowered to 5 μg/dL and effective 14 May 2021, the blood lead reference value for intervention was set at 3.5 μg/dL [22]. By today’s standards, the childhood blood lead concentrations for the CLS are significantly elevated, however, at the various timepoints of the study, the majority were considered typical concentrations below the “action level” for the time of assessment. (The work of the CLS provided key data towards lowering blood lead concentration reference levels for children). 

**Figure 1:**
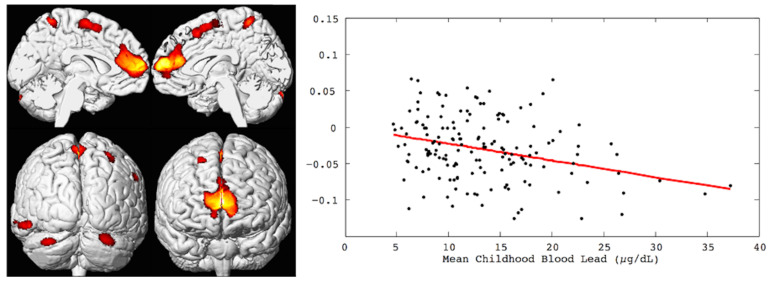
Left, the brain renderings illustrate the regional gray matter volume loss inversely associated with the mean childhood blood lead concentrations (derived from 23 assessments at ages 3 to 78 months) with covariates from the Cincinnati Lead Study. The population imaged includes 157 adults ages 19 to 24 years imaged at 1.5 Tesla. Right, the linear dose-dependent relationship of individual regional brain volume plotted with the mean childhood blood lead concentration within the medial frontal cluster. The multiple linear regression model accounts for age at time of imaging and birth weight.

CLS participants were between the ages of 19 and 24 years for the initial round of multimodal neuroimaging studies conducted at 1.5 Tesla. High-resolution, three-dimensional (3D) anatomical T1-weighted MRI with voxel-based morphometric analyses revealed an inverse, linear dose–effect relationship between mean childhood blood lead concentration and brain volume in specific regions with adjustment for birth weight and age at imaging [7]. Prefrontal cortical areas of lead-associated volume loss involved the medial and the superior frontal gyri, which included the ventrolateral prefrontal cortex as well as the anterior cingulate cortex. Other areas of lead-associated volume loss were in postcentral gyri, the inferior parietal lobule, and the cerebellar hemispheres. Approximately 1.2% of the total gray matter was significantly and inversely associated with the mean childhood blood lead concentration [7]. These findings are illustrated in Figure 1.

**Figure 2:**
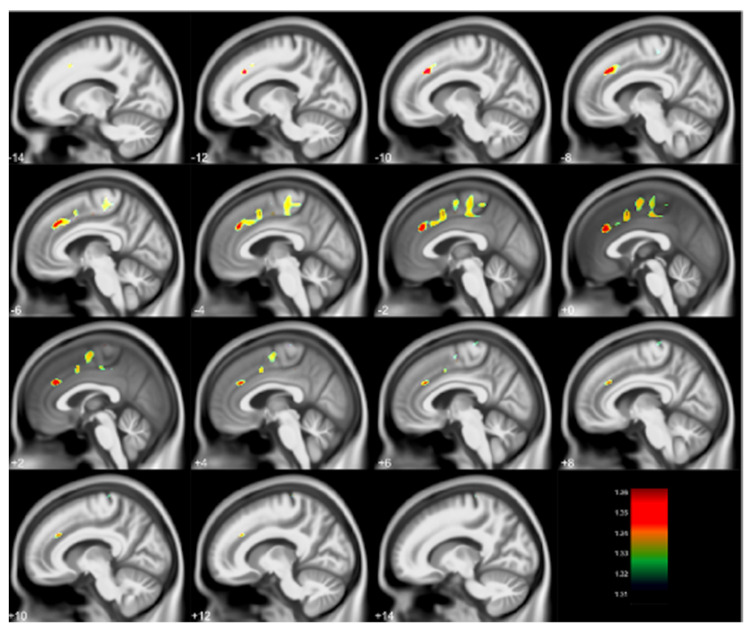
Regional gray matter volume loss inversely associated with childhood blood lead concentrations obtained at age 78 months with covariates from the Cincinnati Lead Study. The population imaged includes 123 adults ages 27–33 years imaged at 3 Tesla. Clusters were corrected for multiple comparisons (FWE = 0.05) using threshold free cluster enhancement. Negative slice numbers = left hemisphere, positive slice numbers = right hemisphere. Color bar represents −log(*p*) value from 1.31 to 1.36.

**Figure 3:**
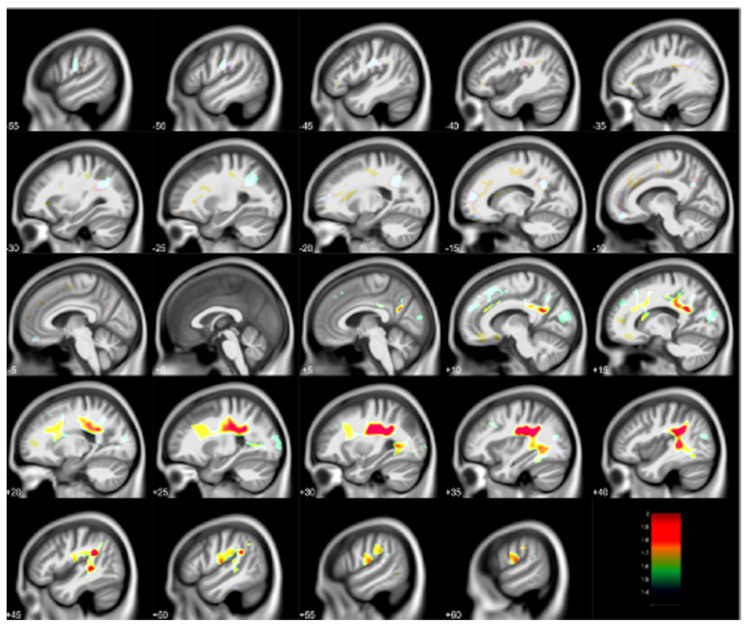
Regional white matter volume loss inversely associated with childhood blood lead concentrations obtained at age 78 months with covariates from the Cincinnati Lead Study. The population imaged includes 123 adults ages 27–33 years imaged at 3 Tesla. Clusters were corrected for multiple comparisons (FWE = 0.05) using threshold free cluster enhancement. Negative slice numbers = left hemisphere, positive slice numbers = right hemisphere. Color bar represents −log(*p*) value from 1.4 to 2.

A second neuroimaging examination was performed with the CLS participants using a 3 Tesla scanner during the visit with participants ages 27–33 years. This work replicated the key frontal gray matter findings and extended it to frontal white matter with improved spatial resolution and statistical modeling [8,9]. Volumes of gray matter regions inversely associated with blood lead concentrations at 78 months included portions of the cingulate cortex, medial frontal gyrus, superior frontal gyrus, paracentral lobule, and supplementary motor area. These findings are shown in Figure 2. The volumes of white matter about the orbital frontal gyrus, cingulate, paracentral lobule, pre- and post-central gyri, superior temporal gyrus, as well as deep white matter structures within the temporal and parietal lobes were inversely associated with blood lead concentrations at 78 months. These findings are illustrated in Figure 3. These associations between childhood blood concentrations with volumetric reductions were subsequently linked with increased adult psychopathy measures and numbers of criminal arrests [8,9]. 

Higher mean childhood blood lead concentrations adjusted for the impact of age at time of imaging and FSIQ were associated with lower metabolite concentrations using quantitative single voxel MRS in the left frontal white matter, left parietal white matter, left basal ganglia, left cerebellar hemisphere, and vermis [10]. For gray matter structures, increases in the mean childhood blood concentration correlated with a decline of NAA and Cr concentrations in the basal ganglia, a reduction of NAA and choline (Cho) concentrations in the cerebellar hemisphere, and a reduction of glutamate and glutamine (Glx) concentration in the vermis. For white matter regions, reductions of Cho and Glx concentrations in parietal lobe and Cho concentration in frontal lobe were observed with increasing mean childhood blood lead concentrations. Surprisingly, there was no decrease of NAA within the frontal cortex. This may reflect neurons within the frontal cortex affected by lead suffered apoptosis, with residual cortex within this region having viable residual neurons yielding appropriate NAA levels. This would then suggest that other sampled brain regions did not suffer significant volume loss, but possibly damage or reorganization in a diminished fashion with NAA and/or Cho changes [10].

**Figure 4:**
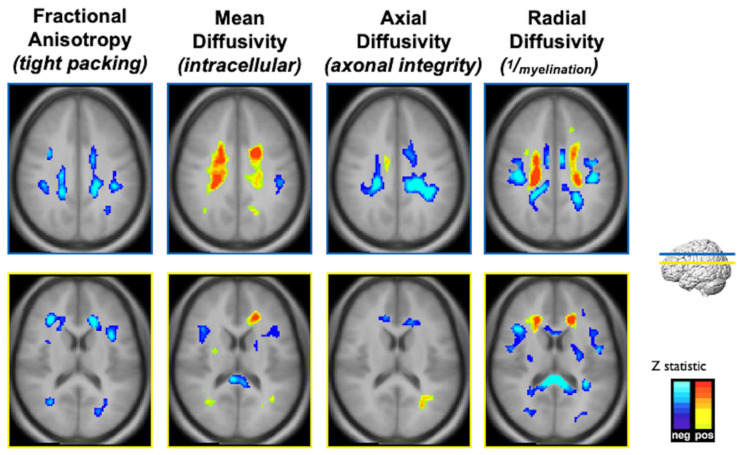
Composite Z-maps showing voxel-wise associations between mean childhood blood lead concentration and fractional anisotropy, mean diffusivity, axial diffusivity and radial diffusivity, respectively, adjusted for significant covariates from left to right for two slice locations, blue at the level of the corona radiata, yellow at the level of the lateral ventricles. The minimum Z statistic was based upon Monte Carlo simulations and corresponds to a two-tailed, corrected voxel-wise *p* value of <0.05. Blue colors represent inverse associations, yellow-red colors represent direct associations.

With these metabolic changes in white matter regions, the long-term impact of childhood lead exposure on white matter integrity was studied using diffusion tensor imaging (DTI) with adjustment for confounding variables [11]. The results are summarized in Figure 4. An inverse association between fractional anisotropy (FA) and mean childhood blood lead concentrations was diffusely observed scattered among white matter regions, including the internal capsule, anterior and superior corona radiata. Mean diffusivity (MD) values exhibited both inverse and direct correlations with mean childhood blood lead concentrations; the primary inverse relationship was observed in the corpus callosum, while the primary direct relationship was noted in the superior corona radiata. Axial diffusivity (AD) values within the anterior and superior corona radiata were inversely correlated with mean childhood blood lead concentrations, with a small focus of direct association found within the body of the corpus callosum. Finally, both inverse and direct correlations between radial diffusivity (RD) and mean childhood blood lead concentrations were observed with the primary inverse relationship observed in the corpus callosum, and internal capsule, while the direct relationship noted in the superior corona radiata. Two patterns of injury were observed, both of which provide evidence supporting altered structural connectivity. The first is a classical injury pattern (low FA, high MD, low AD, high RD) indicating involvement of both the axonal and myelin constituents of white matter. The corona radiata is a later myelinating structure requiring prolonged maturation of cortical connections. The fetal and childhood lead exposure for this later-developing structure produced adverse effects to both axonal and myelin units. In contrast, the second is an unusual pattern (low MD, low RD) with the corpus callosum and internal capsule demonstrating evidence of altered, and possibly increased myelination. The corpus callosum and internal capsule, particularly the posterior limb, are structures whose myelination begins in utero and is usually completed within the first year of postnatal life. The relative early completion of myelination may afford axonal protection and some measure of adaptation for these structures in response to lead exposure during development. 

**Figure 5:**
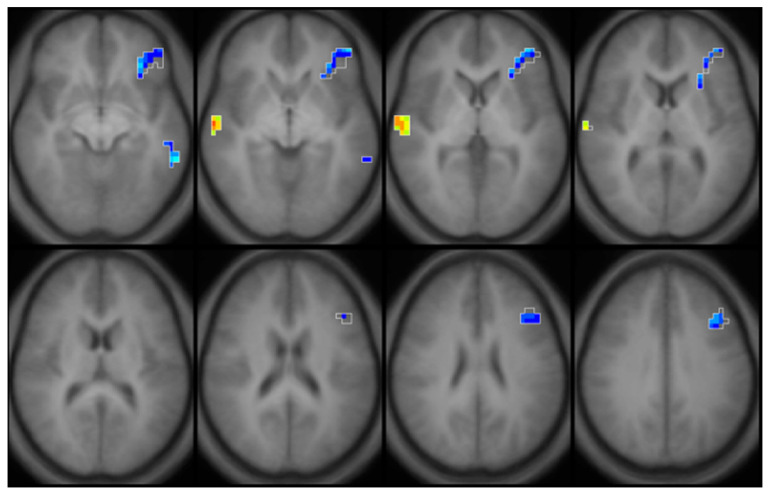
Composite partial R map with covariates. Regions in *blue* illustrate an inverse association (Partial R = −0.328, *p* = 0.39), regions in *yellow* (Partial R = 0.354, *p* = 0.025) illustrate a direct association between brain activation and mean childhood blood lead concentrations in young adults (ages 20–23, *n* = 42) from the Cincinnati Lead Study, with adjustment for birth weight and marijuana usage.

Adults recruited from the CLS underwent a functional (fMRI) protocol employing a semantic language task [12]. For the left inferior and middle frontal gyri (Brodmann areas (BA) 46, 47, 9, 10), adjacent to the region including the traditional Broca’s area, and the left middle temporal gyrus (BA21), a region involved in language and auditory processing, activation levels were inversely correlated to mean childhood blood lead concentrations with adjustment for birth weight and marijuana usage. This finding indicated a damaging effect to the traditional language areas. Activation levels of the right superior/middle temporal gyri (BA22, 42) contralateral to the traditional Wernicke’s area, a region regarded as responsible for speech perception, are positively correlated with mean childhood blood lead levels with covariate adjustment. This finding suggested a necessary compensation mechanism for recruiting new areas, contralateral to typical Wernicke’s area given the reduced “capacity” in the left hemisphere associated with early childhood lead absorption. These findings are illustrated in Figure 5. The neuroplasticity observed is consistent with the established understanding about language function reorganization, i.e., the brain can compensate for injury to regions that are structurally and functionally dedicated to language by recruiting support from other cortical regions. However, increased compensation does not necessarily yield equivalent clinical performance.

#### 2.1.3. Neuroimaging of the Dunedin New Zealand Study Evaluating the Effects of Childhood Lead Exposure

Starting in 1972, the Dunedin Study setup a population representative birth cohort in New Zealand. Anatomical and diffusion MRI was acquired for participants at age 45 years who previously had blood lead concentrations assessed at 11 years of age (*n* = 564 members representing 57% of the original cohort, 54% male) [14]. The mean blood lead concentration at 11 years was 10.99 (SD 4.63) μg/dL. After adjusting for covariates, each 5-μg/dL higher childhood blood lead concentration was significantly associated with 1.19-cm (cm)^2^ smaller cortical surface area, 0.10-cm^3^ smaller hippocampal volume, lower global fractional anisotropy, and a BrainAGE index 0.77 years older than expected at age 45 years. However, the study authors found no statistically significant associations between blood lead concentration and log-transformed white matter hyperintensity volume or mean cortical thickness [14]. 

#### 2.1.4. The ABCD Study, Morphometry and Lead Exposure Risk 

The ABCD study is an ongoing longitudinal study with multimodal neuroimaging acquired in 9- and 10-year-old children from across the United States with planned follow-up study visits as they become adolescents. As the study did not have any individual childhood blood lead concentration assessments, they determined neighborhood lead risk by modeling data pertaining to ages of homes and poverty rates from an individual’s census tract. Using 3D anatomical T1-weighted MRI, Marshall et al., investigated the relationships between the derived lead exposure risk with subcortical volumes, cortical surface areas, cortical volumes, subcortical volumes, and performance on the NIH Toolbox cognitive assessments [15,16]. Children who lived in neighborhoods with greater risks of environmental lead exposure demonstrated smaller volumes of the mid-anterior, central, and mid-posterior corpus callosum. These callosal volumes were associated with poorer performance on cognitive tests measuring language and processing speed. 

The analyses also found that children within the low-income group demonstrated a 5.6% reduction in cortical volume relative to the high-income group. In comparison, the mean cortical volume for participants living in the highest neighborhood lead-risk tracts was 9.6% smaller for those in the low-income group than in the high-income group. Vertex maps generated by subtracting the means of participants living in high-lead-risk census tracts from the means of those living in low-lead-risk census tracts (lead risk ≤ 3), illustrated global decreases in cortical surface area and volume across the entire cortex for children classified in the low-income group relative to those in the high-income group. Further analyses reported by Karcher et al. found that structural volumes mediated between 11% and 25% of the associations found with poverty, neighborhood safety perception, and lead exposure risk with psychotic-like experiences for participants in the ABCD study [23].

#### 2.1.5. Fetal Brain Functional MRI and Lead Exposure

Thomason et al. examined human fetal brain functional MRI data in a cohort (*n* = 26) to determine neural network connectivity differences between lead-exposed and lead-naive fetuses between 23- and 40-weeks gestation [17]. Prenatal lead exposure was extracted from newborn bloodspots. Those below the detection limit of 1.0 μg/dL were classified as lead-naïve (*n* = 13), while those above as lead-exposed ((*n* = 13) mean lead 2.43 μg/dL, range 1–11 μg/dL). Groups were matched for gestational age at birth, gestational age at fetal scan, birth weight, and factors pertaining to fMRI data quality and motion. Neural connectivity patterns differed as fetuses that were classified as lead-naïve demonstrated stronger age-related increases in cross-hemispheric connectivity, such as in the bilateral insular cortices, while the lead-exposed fetuses showed stronger age-related increases in posterior cingulate cortex to lateral prefrontal cortex connectivity, as in the superior frontal gyrus. The findings suggested that the frontoparietal control network and the default mode network enhanced connectivity within the lead-exposed group could contribute to later developmental psychopathology and executive dysfunction.

### 2.2. Manganese Characteristics and MRI

Due to its paramagnetic properties, the presence of Mn in the human brain can be quantified with T1-weighted anatomical MRI [24]. Mn shortens the water proton T1 relaxation rate in brain tissue, producing hyperintense signal within sub-structures where Mn is stored. 

#### 2.2.1. Canadian Children, Manganese in the Water, Pallidal Signal Indices and Morphometry

Bouchard et al. ([25]) studied 362 children from Quebec, Canada, and showed a negative association between Mn concentration in drinking water and FSIQ, as well as poorer performance of memory, attention, and motor functions [26]. Dion et al. evaluated a subset of this Canadian cohort examining relaxation properties [27]. Quantitative T1 and T2 mapping sequences were obtained from 10 children with long-term exposure to higher water Mn (median 145 μg/L) and 13 children as age-matched controls with lower water Mn concentrations (median 0.9 μg/L). Three metrics were compared between groups: (1) The signal intensity within the globus pallidus relative to subcortical frontal white matter (known as the standard pallidal index (PI)); (2) the signal intensity within the globus pallidus relative to pericranial muscles; and (3) calculated T1 relaxation time. Children exposed to higher water Mn concentrations had lower PI referenced to pericranial muscles and higher T1 relaxation time compared to those in the lower Mn water concentration group. The standard PI failed to show any differences between the higher and lower water Mn groups [27].

Lao et al. performed both a traditional volume-based analysis and a multivariate tensor-based morphometry (mTBM) analysis of basal ganglia subnuclei from brain T1 weighted anatomical MRI of the same children in higher and lower water Mn groups described by Dion et al. [27,28]. Traditional volume-based analyses demonstrated only a trend for slightly larger volumes of the putamen, globus pallidus, and caudate in the higher Mn water group compared with the lower. However, surface-based morphometry, after structure-wise multiple comparisons correction, revealed significant differences between the exposure groups in the bilateral putamen with similar trends for the bilateral caudate and globus pallidus. Reduced motor performance in the Santa Ana Pegboard test was also significantly correlated with regional enlargement in anterior ends of the putamen, globus pallidus and caudate, which were the same areas identified in the mTBM comparisons between Mn exposure groups [28].

#### 2.2.2. Italian Adolescents, Manganese, and Resting State Functional Magnetic Resonance Imaging

De Water et al. investigated the associations between prenatal, early postnatal, and childhood dentine Mn concentrations and intrinsic functional connectivity (iFC) for adolescents enrolled in a longitudinal cohort from northern Italy [29]. The study team estimated prenatal, early postnatal, and childhood Mn concentrations in deciduous teeth using laser ablation-inductively coupled plasma-mass spectrometry. Dentine Mn concentrations (Mn:Calcium ratio) were highest during the prenatal period and significantly diminished throughout childhood. Resting state functional MRI (rs-fMRI) from 14 adolescents (12–18 years; 6 girls) was post-processed with seed-based correlation analyses featuring six subcortical seeds (pallidum, putamen, left, and right caudate) and one cortical seed (bilateral middle frontal gyrus) from Harvard-Oxford atlases. With linear and quadratic correlations between log-transformed Mn concentrations and seed-based iFC (Bonferroni-corrected), controlling for either socio-economic status, sex or age, the study reported postnatal Mn concentrations were associated with increased iFC between the middle frontal gyrus and medial prefrontal cortex, however, decreased iFC between the right putamen and pre- and postcentral gyrus. Summarizing the preliminary work, the study found early postnatal Mn concentrations are associated with increased iFC within cognitive control brain areas, but decreased iFC between motor areas in adolescents [29].

## 3. Air Pollutants

Air pollution arises from a mixture of natural and anthropogenic sources. Emissions from vehicles, home heating oil and natural gas, by-products of manufacturing and power generation, and fumes from chemical production are the primary sources of human-made air pollution. Numerous studies have linked air pollution to an assortment of adverse health outcomes including premature birth, low birth weight, respiratory diseases, cardiovascular diseases, and cancers. Emerging science also links air pollution effects to the brain with conditions such as attention-deficit hyperactivity disorder related behavior, autism, and neurodegenerative diseases including Parkinson’s disease, Alzheimer’s disease, and other dementias. 

Traffic related air pollution (TRAP) consists of sulfur oxides, nitrogen oxides, ground-level ozone, many carbon-based chemicals, polycyclic aromatic hydrocarbons (PAH), volatile organic compounds, and particulate matter (PM). Air pollution modelling builds upon data from emission monitoring programs for a region while combining information about terrain and meteorological conditions. Primary pollutants are emitted into the atmosphere directly while others are formed indirectly from chemical reactions within the atmosphere. The studies highlighted in the text and summarized in Table 2 incorporate different spatiotemporal models with distinct input variables. At this point, only general comparisons can be made as the exposure models are quite complex and when compared with imaging outcomes often include differing confounding variables. Moreover, the intrinsic spatial resolution differs across exposure models. Finally, these models often assume that a child’s residential address is the location where most of the child’s time is spent and thus, the most representative index of exposure occurs there.

### 3.1. Pre- and Postnatal Polycyclic Aromatic Hydrocarbons and Morphological MRI in Urban Children

Peterson and colleagues evaluated 40 urban children from New York City with anatomical MRI between ages 7 and 9 years [30]. Pre- and postnatal PAH exposures were compared with morphological measures that indexed local volumes on the surface of the brain and the white matter surface upon removal of cortical gray matter. The study did not find an association between individual PAH exposure during the third trimester of pregnancy and any measure of cortical thickness. However, they reported an association between higher PAH exposure during the third trimester of pregnancy and a lower white matter surface, almost exclusively to the left hemisphere of the brain. The study authors observed prenatal exposure to PAH air pollutants contributed to slower processing speed, attention-deficit/hyperactivity disorder symptoms, and externalizing problems in urban children and disrupted the development of left hemisphere white matter, whereas postnatal PAH exposure contributed to additional disturbances in the development of white matter in dorsal prefrontal regions [30].

### 3.2. Barcelona Schools Study of Polycyclic Aromatic Hydrocarbons

Mortamais et al. estimated outdoor and indoor PAH exposures in Spanish schools for comparison with basal ganglia and whole brain volumes in children (*n* = 242, 49% female, from 35 schools) [31]. An increase of approximately 70 pg/m^3^ in the concentration of indoor or outdoor benzo[a]pyrene in the school environment was significantly associated with a reduction corresponding to almost 2% of the mean caudate nucleus volume in the study population of children aged 8 to 12 years adjusted for intracranial volume, age, sex, maternal education and socioeconomic vulnerability index at home [31]. Benzo[a]pyrene levels were below the legislative annual target level established by the European Union.

### 3.3. Morphological MRI of Children and Adolescents from The Netherlands Exposed to Air Pollution

Guxens et al. evaluated if air pollution exposures during fetal life altered brain morphology and if the alterations mediate the exposures relationships with cognitive function in *school-age children* [32]. Air pollution modeled by the team using land-use regression models included nitrogen dioxide (NO_2_), coarse particles (difference between particles with aerodynamic diameters < 10 μm and <2.5 μm), fine particles (particles with aerodynamic diameters < 2.5 μm), and absorbance of fine particles (proxy for elemental carbon). Children (*n* = 783, ages 6–10 years) from a population-based birth cohort in Rotterdam, The Netherlands, born between 2002 and 2006, and exposed to higher PM concentrations during fetal life demonstrated thinner cortex in several brain regions of both hemispheres, with more associations found in the right hemisphere. The team determined the cerebral cortex of the precuneus region in the right hemisphere was 0.045 mm (mm) thinner for each 5-μg/m^3^ increase in PM_2.5_. The reduced cerebral cortex in the precuneus and rostral middle frontal regions partially mediated the association between exposure to PM and impaired inhibitory control. However, exposures were not associated with global brain volumes [32]. Their models were adjusted for parental educational levels, monthly household income, parental countries of birth, parental ages, maternal prenatal smoking, maternal prenatal alcohol use, parental body mass indices and heights, maternal parity, family status, maternal psychological distress, maternal FSIQ, and child gender, age, and genetic ancestry. Lubczynska et al. also using participants from the Rotterdam cohort, however, imaged as *pre-adolescents* (*n* = 3133, 9–12 years old) with additional types and greater exposure details of air pollutants modeled during pregnancy and childhood, also found no associations with exposures and global brain volumes [33]. However, the study found when using single pollutant analyses, multiple associations between higher pregnancy and childhood pollutant concentrations with smaller corpus callosum, smaller hippocampus, larger amygdala (pregnancy only), and larger cerebellum (pregnancy only). Higher gestational exposure to air pollutants was associated with smaller cortical thickness in the right postcentral gyrus, right rostral middle frontal gyrus, and the left lingual gyrus, while higher childhood exposure was associated with predominantly larger cortical surface area within the right pre- and post-central gyri, the left precuneus and calcarine cortex [33]. Models were adjusted for parental educational levels, household income, parental countries of birth, parental ages, maternal prenatal smoking, maternal alcohol consumption during pregnancy, maternal parity, marital status, and parental psychiatric symptoms, parental heights and body mass indices, maternal intelligence quotient, child’s genetic ancestry, child’s gender and child’s age at the scanning session. Subcortical grey matter, corpus callosum, cerebellum, and subcortical brain volumes were additionally adjusted for intracranial volume.

### 3.4. Morphological MRI and Magnetic Resonance Spectroscopy of Cincinnati Children Exposed to Traffic Related Air Pollution

**Figure 6:**
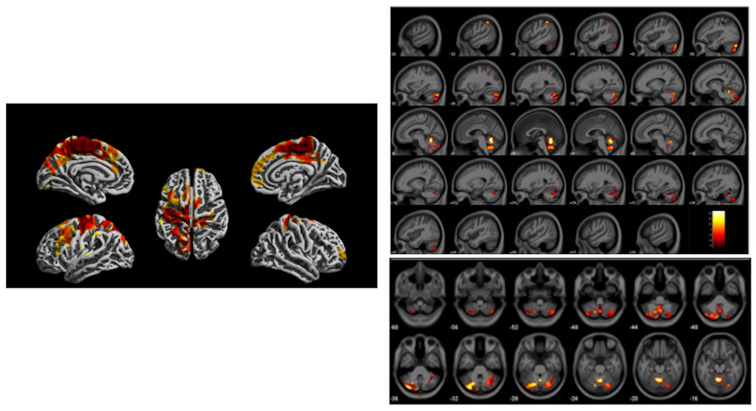
Imaging findings acquired from a 3 Tesla scanner at age 12 years from the Cincinnati Childhood Allergy and Air Pollution Study (CCAAPS) comparing high and low groups based upon estimated element carbon attributable to traffic (ECAT) exposure in the first year of life. Left, regions illustrated in red and yellow on brain renderings oriented with different views demonstrate regional reduced cortical thickness. Right, regional gray matter volume loss illustrated across the brain in a sagittal view (top) and axial view showing cerebellar region (bottom) associated with the high ECAT group compared to the low ECAT group. Clusters were corrected for multiple comparisons using threshold free cluster enhancement with a familywise error rate of *p* < 0.05. Color bar represents -log (*p*) value from 1.4 to 2.6.

Beckwith et al. examined whether childhood TRAP exposure is associated with regional differences in brain volume and cortical thickness among children enrolled in a longitudinal cohort study based in Cincinnati, Ohio USA [34]. Anatomical T1-weighted MRI from a nested subset of 12-year-old participants (*n* = 135) characterized with either higher or lower levels of TRAP exposure during their first year of life was analyzed with voxel-based morphometry to examine group differences in regional brain volume, and with separate analyses, changes in cortical thickness. Smaller regional gray matter volumes were found in the left pre- and post-central gyri, the cerebellum, and inferior parietal lobe of participants in the higher TRAP exposure group relative to participants with lower exposure. Reduced cortical thickness was observed in participants with higher exposure relative to those with lower exposure, primarily in sensorimotor regions of the brain including the pre- and post-central gyri and the paracentral lobule, but also within the frontal and limbic regions [34]. These findings are shown in Figure 6. Age at MRI examination, birth weight, participant FSIQ, census tract deprivation status at birth, and race were selected for inclusion in the final model. Total intracranial volume was also included as a covariate for volumetric analyses.

**Figure 7:**
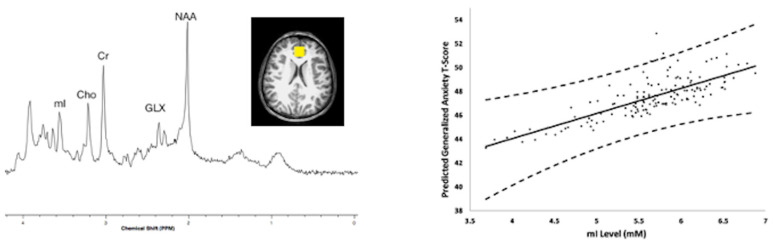
Magnetic resonance spectroscopy was acquired using a 3 Tesla scanner from the Cincinnati Childhood Allergy and Air Pollution Study (CCAAPS) cohort at age 12 years. Left, representative short echo spectrum from a CCAAPS participant with notations for neurochemicals, N-acetylaspartate (NAA), Glutamate and glutamine (GLX), creatine and phosphocreatine (Cr), cholines (Cho), myo-inositol (mI) and an insert of a representative voxel location shown in yellow within the perigenual anterior cingulate cortex for the 8 cubic centimeter (2 cm per side) positioned on an axial T1-weighted image. Higher myo-inositol concentrations were observed for participants with higher estimated element carbon attributable to traffic (ECAT) in the previous year of life. Right, the relationship between myo-inositol concentration and child reported generalized anxiety T-scores obtained from a generalized anxiety subscale of the Spence Children’s Anxiety Scale (SCAS) and adjusted for covariates at age 12 years.

In this same cohort, Brunst et al. reported participants with exposure to high levels of TRAP within the year prior demonstrated higher myo-inositol concentrations within the medial frontal lobe and anterior cingulate cortex compared to participants with lower TRAP exposure [35]. Recent higher TRAP exposure and increased myo-inositol levels were also significantly associated with increased generalized anxiety symptoms with 12% of the total effect mediated by myo-inositol concentrations [35]. These findings are shown in Figure 7. Covariates included child’s race, household income, maternal age at study enrollment, caregiver depression assessed by the Beck Depression Inventory, relational frustration pertaining to the parent-child relationship (assessed by the Parent Relationship Questionnaire), and serum cotinine levels.

### 3.5. The ABCD Study, Morphological MRI and PM_2.5_ Exposure

Cserbik and colleagues examined the cross-sectional associations between concurrent PM_2.5_ exposures, hemisphere- and regional-specific differences in brain morphometry and measures of cognition in 10,343 children (ages 9–10 years) who participated in the ABCD Study across the United States [36]. All models were adjusted for age, sex, ethnicity, neighborhood quality, parental higher education, total family income, parental employment status, imaging device manufacturer, handedness and intracranial volume for volumetric measures, including random intercepts for ABCD site and familial relationship. Models were scaled for PM_2.5_ using a fixed increment of 5 μg/m^3^. Annual residential PM_2.5_ exposure was associated with hemispheric-specific differences in gray matter across cortical regions of the frontal, parietal, temporal, and occipital lobes as well as subcortical and cerebellum brain regions. There were *hemispheric*-specific associations between PM_2.5_ exposures and cortical surface area in 9/31 regions, cortical thickness in 22/27 regions; and volumes of the thalamus, pallidum, and nucleus accumbens. The analyses revealed regional specific associations between PM_2.5_ exposure and cortical thickness of the frontal lobe, temporal lobe, and basal ganglia as well as differences in the directionality of associations by hemisphere, some smaller, others larger. An increase of 5-μg/m^3^ was associated with differences in cortical thickness on the magnitude ranging from 0.01 to 0.04 mm, suggesting that continual exposure to PM_2.5_ during childhood and adolescence could substantially impact an individual’s brain growth trajectories with potentially lifelong consequences. However, the team found neither significant associations between PM_2.5_ and task performance on individual measures of cognition nor evidence that sex moderated the observed associations [36].

## 4. Pesticides

Pesticides encompass a variety of chemicals including disinfectants, fungicides, herbicides, insecticides, and poisons for controlling mice and rats. These chemicals can exist in our food, homes, workplaces, schools, and hospitals. Pesticide exposures are increasingly linked to adverse neurodevelopmental effects in children.

### 4.1. Prenatal Chlorpyrifos Exposure and Morphometric MRI in New York Children

Rauh and colleagues investigated associations between prenatal chlorpyrifos (CPF) exposure and brain morphology using T1-weighted anatomical MRI in 40 children (ages 5–11 years) recruited from an urban, New York City, community-based cohort [37]. Classifying exposure groups by umbilical cord blood CPF concentrations, twenty high-exposure children were compared with 20 low-exposure children for morphological features. After adjusting for age and height, brain sizes were not significantly different between the groups. However, high-exposure children showed frontal and parietal cortical thinning and revealed an inverse dose–response relationship between CPF and cortical thickness. Children in the higher exposure group demonstrated regional enlargements of the cerebral surface area in the superior temporal, posterior middle temporal, and inferior postcentral gyri bilaterally, and in the superior frontal gyrus, gyrus rectus, cuneus, and precuneus along the mesial wall of the right hemisphere [37]. These regions support cognitive and behavioral processes including attention, receptive language, social cognition, reward, emotional, and inhibitory control.

### 4.2. Prenatal Organophosphate Exposure and Morphometric MRI in Generation R Study Subset

Mother–child pairs (*n* = 518) participating in the Generation R Study, a population-based birth cohort from Rotterdam, the Netherlands, were studied to determine whether maternal organophosphate (OP) pesticides were associated with child brain morphology [38]. Maternal urine concentrations of OP pesticides collected throughout pregnancy were analyzed for 6 dialkylphosphates (DAPs) including 3 dimethyl (DM) and 3 diethyl (DE) alkyl phosphate metabolites. Anatomical T1-weighted MRI and DTI were acquired when children were between the ages of 9 and 12 years. Brain volumes, surface-based cortical thickness and cortical surface area, and diffusion metrics, FA and MD, were compared with prenatal OP exposures. DM and DE metabolite concentrations were not associated with brain volumes, cortical thickness, and cortical surface area. However, a ten-fold increase in averaged DM metabolite concentrations across pregnancy was associated with lower FA and higher MD. Similar associations were observed for DE concentrations [38]. The models were adjusted for age of child at time of imaging and sex, and maternal age, ethnicity (Dutch, other-western, and non-western), education, income, marital status, alcohol consumption during pregnancy, body mass index, parity, and smoking during pregnancy.

### 4.3. Prenatal Organophosphate Exposure and Motor Inhibition Functional Magnetic Resonance Imaging

Binter et al. evaluated the association of prenatal OP exposure on inhibitory control employing an fMRI motor inhibition (Go/No-Go task) paradigm [39]. Ninety-five children (ages 10–12 years) from a longitudinal birth cohort completed the fMRI and had maternal OP concentrations available for comparisons. OP exposure was assessed by measuring six DAP metabolites in the urine of women in early pregnancy (<19 weeks gestation). Concentrations were categorized into levels of exposure: low (reference), moderate, or high. Task performance was characterized by average response latency, commission rate, and composite performance score. The models were adjusted for maternal fruit/vegetable consumption and the duration of breastfeeding. Moderate levels of DAP were associated with a decreased commission rate indicating improved performance. Increasing levels of DM and DE were associated with decreased brain activity in the left inferior and bilateral superior frontal regions during successful inhibition. The investigators did not observe any differential activation related to inhibitory demands [39].

## 5. Environmental Tobacco Smoke

Environmental tobacco smoke (ETS), also known as secondhand smoke, refers to the chemical mixture of smoke produced by burning tobacco products and exhaled smoke from active smokers. Exposure to involuntary ETS causes numerous health problems in infants and children, including asthma attacks, respiratory infections, ear infections and sudden infant death syndrome. For children, the indoor home environment from a smoker in their home is the most common source. Cotinine, the major metabolite of nicotine, is a frequently employed biomarker for assessing recent ETS exposure due to its high sensitivity and specificity, and ease of measurement by sampling urine, saliva, hair, or serum.

### 5.1. ETS, Multimodal MRI, Cognitive Control Circuitry in Urban Children

To evaluate the effects of ETS exposure, Margolis et al. recruited from a prospective longitudinal urban birth cohort, selecting non-smoking mothers and their children ages 7–9 years [40]. Prenatal maternal and postnatal child urine samples were analyzed for cotinine concentrations. Those with concentrations between 0.05 and 0.99 ng/mL were regarded as non-exposed to ETS in comparison to those with concentrations between 1 and 16 ng/mL considered ETS-exposed. The children completed anatomical T1 weighted MRI (*n* = 41, 24 ETS-exposed) and fMRI using the Simon Spatial Incompatibility Task (*n* = 30, 10-exposed). Compared to non-exposed children, prenatally ETS-exposed children had smaller left and right thalamic and inferior frontal gyrus (IFG) volumes, and increased activation in the IFG during the resolution of cognitive conflict measured with task fMRI. Reduced thalamic volume was associated with increased IFG activation and attention problems. Post-hoc analyses of postnatal exposure were not associated with brain measures when prenatal exposure was included in the model. Margolis et al. suggested that maternal ETS exposure during pregnancy posited deleterious effects on the structure and function of cognitive control circuitry which impacted attentional capacity in school-age children [40].

### 5.2. ETS, Go-No-Go fMRI

Using an event-related fMRI design, Bennett et al. compared the brain function of ETS-exposed (*n* = 7) and unexposed (*n* = 11) 12-year-olds during a Go/No-Go response inhibition task [41]. Exposure grouping was derived from maternal and child self-report. ETS-exposed children showed greater activation within many regions, especially the left frontal, right occipital, and bilateral temporal and parietal lobes compared with activation patterns in unexposed children. However, children not exposed to ETS showed greater activation in the cerebellum and the occipital lobe. ETS exposure was associated with a different pattern of brain activation among children in early adolescence while controlling for potential confounding factors [41].

## 6. Flame Retardants

Flame retardants are chemicals applied to consumer and industrial products such as home, school and office furnishings, electronics, building materials, and other materials to prevent or impede the growth of fire. These chemicals can contaminate the air, water, and soil during production, leak from products into dust to be handled and inhaled and become airborne with electronic waste. Emerging evidence suggests these chemicals adversely impact health through endocrine and thyroid disruption, which can also affect fetal and child development, neurologic, and immune function. Further studies indicate reproductive toxicity and cancer linkage to flame retardants. These chemicals are classified into categories based on whether they contain boron, bromine, chlorine, metal, nitrogen, or phosphorus.

de Water and colleagues used rs-fMRI to examine associations between prenatal polybrominated diphenyl ether (PBDE) concentrations measured in maternal serum and intrinsic functional network organization in 5-year-old children (*n* = 34), adjusting for sex, maternal education, and home environment [42]. Children with higher prenatal PBDE serum concentrations showed increased global efficiency of brain areas involved in memory (hippocampus) and visual attention (lingual gyrus, inferior and superior occipital gyrus, posterior inferior and middle temporal gyrus), and decreased global efficiency of brain areas involved in sensorimotor functions (precentral gyrus) and visual (middle occipital gyrus), and auditory processing (superior temporal gyrus). Children with higher prenatal PBDE serum concentrations showed more parent-reported executive function problems, including poor inhibition and shifting. Children who showed increased global efficiency of brain areas involved in visual attention, specifically the lingual gyrus and inferior occipital gyrus presented with more parent-reported executive function problems [42].

## 7. Summary

The importance of neuroimaging studies of known and suspected neurotoxicants in children is quite significant. Fetuses, infants, and children are among the most vulnerable members of our society. Their exposure to neurotoxicants in the womb and throughout childhood will have life-long impacts on their individual health and well-being as well as society at large. Most of the reviewed articles featured gestational exposures as opposed to associations with concurrent exposures. These studies typically employ longitudinal cohorts to examine multiple timepoints. Often when the maternal exposure is the only timepoint reported, it holds the only significant or the strongest association. However, hand-to-mouth behavior and proximity to the floor increases the potential of children to be exposed to various neurotoxicants postnatally. Conducting epidemiological studies in pregnant women and pediatric populations is difficult and expensive, even more so with the addition of MRI. Ethical concerns must also be managed. Reporting results to families, especially the unknown meaning of some observations, and incidental findings are among the challenges. Despite the relatively small number of studies combining environmental epidemiology and advanced neuroimaging, this review illustrates that indeed there is growing evidence that environmental neurotoxicants exert structural, organizational, and functional changes in the pediatric brain.

These types of investigations are reliant on strong environmental epidemiology and neuroimaging expertise to achieve accurate conclusions. Many studies cited in this review are using standard neuroimaging post-processing approaches, such as FreeSurfer [13] for morphological measures of brain volume, cortical thickness, and surface area. Such standardization will help build consensus across cohorts and investigators to move from associations to stronger evidence supporting causality.

It is important to recognize study design features for the types of research described within this review. Most studies employ a multivariable linear regression statistical approach to search for associations with adjustments for confounding variables. The summary of each published article attempted to detail the confounding variables related to imaging outcomes along with exposures. The selection of covariates is limited by prior research knowledge of them, availability of covariates, and how to appropriately model them especially with limited sample sizes. The Generation R and ABCD Study investigations are beginning to address the issue of larger sample sizes. Most studies examine single classes of known and suspected neurotoxicants. In the real world, children are exposed to multiple known and suspected neurotoxicants during gestation and postnatally. The field of environmental epidemiology is actively searching for optimal methods for simultaneously examining mixtures over time. Historically, neuroimaging investigations compare disease and healthy populations. The statistical approaches for blending the fields will need continued refinement.

## Figures and Tables

**Table 1:** Summary of neuroimaging findings associated with increased lead exposure.

Exposure Assessment	Imaging Method	Age at Imaging	Key Findings	Reference
Childhood Blood	MRS	Child	Reduced NAA/Cr in middle frontal cortex	Trope et al. [4,5]
Childhood Blood	MRI: SPM, VBM [6]	Adult	Reduced frontal cortex and white matter volumes	Cecil et al. [7]
Childhood Blood	MRI: SPM, VBM [6]	Adult	Reduced frontal cortex and white matter volumes	Beckwith et al. [8,9]
Childhood Blood	MRS	Adult	Reduced metabolite concentrations	Cecil et al. [10]
Childhood Blood	DTI	Adult	Two disparate patterns of DTI metrics with injury and compensation	Brubaker et al. [11]
Childhood Blood	Task fMRI	Adult	Two disparate patterns of activation with injury and compensation using verb generation task	Yuan et al. [12]
Childhood Blood	MRI: FreeSurfer [13]	Adult	Reduced cortical surface area, hippocampus volume	Reuben et al. [14]
Childhood Blood	Diffusion	Adult	Lower FA	Reuben et al. [14]
Childhood Blood	BrainAge	Adult	Older estimated brain age by 0.77 years	Reuben et al. [14]
Lead Risk from Home Age, Income	MRI: FreeSurfer [13]	Child	Reduced cortical volume, cortical surface area, corpus callosum volume	Marshall et al. [15,16]
Newborn Blood Spot	fMRI	Fetus	Different neural connectivity patterns	Thomason et al. [17]

MRS, magnetic resonance spectroscopy; NAA, N-acetyl aspartate; Cr, creatine; MRI, magnetic resonance imaging; SPM, Statistical Parameter Mapping (www.fil.ion.ucl.ac.uk/spm, assessed 28 February 2022); VBM, voxel-based morphometry; DTI, diffusion tensor imaging; fMRI, functional magnetic resonance imaging; FA, fractional anisotropy.

**Table 2:** Summary of neuroimaging findings associated with air pollutants.

Exposure Assessment	Imaging Method	Age at Imaging	Key Findings	Author, Citation
Prenatal, Postnatal PAH	MRI	Child	3rd Trimester PAH with reduced white matter surface	Peterson et al. [30]
Childhood PAH	MRI: FreeSurfer [13]	Child	Reduced caudate nucleus volume	Mortamais et al. [31]
Prenatal NO_2_, PM	MRI: FreeSurfer [13]	Child	Reduced cortex thickness: right hemisphere 0.045 thinner for each 5 mg/m^3^ increase of PM_2.5_	Guxens et al. [32]
Prenatal and Childhood NO_2_, PM	MRI: FreeSurfer [13]	Adolescent	Reduced corpus callosal, hippocampal volumes, cortical thickness; for childhood exposures, larger cortical surface area; for prenatal exposures, larger amygdala and cerebellum volumes	Lubczynska et al. [33]
Early Childhood TRAP	MRI: SPM, VBM [6]	Adolescent	Reduced gray matter volumes, cortical thickness in sensorimotor regions, cerebellum	Beckwith et al. [34]
Childhood TRAP	MRS	Adolescent	Higher myo-inositol concentrations within medial frontal lobe, anterior cingulate cortex	Brunst et al. [35]
Childhood PM_2.5_	MRI: FreeSurfer [13]	Child	Regional associations with cortical surface area, cortical thickness, and volume	Cserbik et al. [36]

PAH, polycyclic aromatic hydrocarbons; NO_2_, nitrogen dioxide; PM_2.5_, particulate matter less then 2.5 microns; PM, particulate matter, TRAP, traffic related air pollution; SPM, Statistical Parameter Mapping (www.fil.ion.ucl.ac.uk/spm, assessed 28 February 2022); VBM, voxel-based morphometry.

## Data Availability

Not applicable.

## Abbreviations

3D	three dimensional
ABCD Study	Adolescent Brain Cognitive Development Study
AD	axial diffusivity
BA	Brodmann area
Cho	cholines
CLS	Cincinnati Lead Study
cm	centimeter
CPF	chlopyrifos
Cr	Creatine
DAP	diakylphosphates
DE	diethyl alkyl phosphate metabolites
DM	dimethyl alkyl phosphate metabolites
DTI	Diffusion Tensor Imaging
ETS	environmental tobacco smoke
FA	fractional anisotropy
fMRI	functional magnetic resonance imaging
FSIQ	Full Scale Intelligence Quotient
Glx	glutamate and glutamine
iFC	intrinsic functional connectivity
IFG	inferior frontal gyrus
MD	mean diffusivity
μg/L	micrograms per liter
μg/dL	micrograms per deciliter
mm	millimeter
Mn	manganese
MRI	magnetic resonance imaging
MRS	magnetic resonance spectroscopy
mTBM	multivariate tensor-based morphometry
NO_2_	nitrogen dioxide
OP	organophosphate pesticides
PAH	polycyclic aromatic hydrocarbons
PBDE	polybrominated diphenyl ether
pg/m^3^	picograms per cubic meter
PI	pallidal index
PM	particulate matter
PM_2.5_	particulate matter less than 2.5 microns
RD	radial diffusivity
rsfMRI	resting state functional magnetic resonance imaging
SD	standard deviation
TRAP	traffic related air pollution
SPM	statistical parameter mapping
VBM	voxel-based morphometry
